# Effects on Automatic Attention Due to Exposure to Pictures of Emotional Faces while Performing Chinese Word Judgment Tasks

**DOI:** 10.1371/journal.pone.0075386

**Published:** 2013-10-04

**Authors:** Huang Junhong, Zhou Renlai, Hu Senqi

**Affiliations:** 1 Beijing Key Laboratory of Applied Experimental Psychology, School of Psychology, Beijing Normal University, Beijing, China; 2 National Key Laboratory of Cognitive Neuroscience and Learning, Beijing Normal University, Beijing, China; 3 Competitive Sport Research Center, China Institute of Sport Science, Beijing, China; 4 Department of Psychology, California State University, Los Angeles, Los Angeles, California, United States of America; University of California, San Francisco, United States of America

## Abstract

Two experiments were conducted to investigate the automatic processing of emotional facial expressions while performing low or high demand cognitive tasks under unattended conditions. In Experiment 1, 35 subjects performed low (judging the structure of Chinese words) and high (judging the tone of Chinese words) cognitive load tasks while exposed to unattended pictures of fearful, neutral, or happy faces. The results revealed that the reaction time was slower and the performance accuracy was higher while performing the low cognitive load task than while performing the high cognitive load task. Exposure to fearful faces resulted in significantly longer reaction times and lower accuracy than exposure to neutral faces on the low cognitive load task. In Experiment 2, 26 subjects performed the same word judgment tasks and their brain event-related potentials (ERPs) were measured for a period of 800 ms after the onset of the task stimulus. The amplitudes of the early component of ERP around 176 ms (P2) elicited by unattended fearful faces over frontal-central-parietal recording sites was significantly larger than those elicited by unattended neutral faces while performing the word structure judgment task. Together, the findings of the two experiments indicated that unattended fearful faces captured significantly more attention resources than unattended neutral faces on a low cognitive load task, but not on a high cognitive load task. It was concluded that fearful faces could automatically capture attention if residues of attention resources were available under the unattended condition.

## Introduction

It has been recognized that the ability to automatically perceive threatening stimuli provides an adaptive advantage in the survival of an organism [1]. Previous studies have found that an observer’s nervous system automatically orients its attention to novel or threatening stimuli such as the negative facial expressions of fear and anger (e.g., [2], [3], [4], [5], [6], [7], ). The orienting response involves automatic attentional mechanisms that are unconscious and stimulus driven in what is often defined as a bottom-up process [4]. Recent behavioral studies have provided empirical evidence of automatic processing of negative emotional stimuli [9], [10], [11], [12], [13], [14].

Several recent studies using functional magnetic resolution imaging (fMRI) and event-related potential (ERP) techniques have also provided neuroscientific evidence that attention to negative facial expressions such as fear, anger, or disgust is processed automatically or unconsciously. The fMRI studies have shown that the activity of the amygdala increased in response to fearful faces in both attended and unattended conditions. Researchers found that in fMRI scans, differential amygdala responses to fearful versus happy facial expressions were influenced by mechanisms of attention and that the amygdala’s activity significantly increased when exposed to potentially threatening stimuli (fearful faces) than when exposed to non-threatening stimuli (happy faces) under conditions of inattention [15], [16].

Consistent with the fMRI findings, a recent ERP study [17] revealed that the amplitude of P2a component of ERPs increased while exposed to negative emotional faces but not to neutral faces under conditions of inattention, indicating that automatic attention was triggered by negative emotional face. Another recent study [18] also found an enhanced P1 component to negative faces. The source localization of this P1 effect indicated increased activity within the anterior cingulate cortex (ACC), providing mechanisms underlying automatic attention orienting towards negative faces. Other ERP studies [19], [20] also showed that exposure to facial expressions elicited the visual mismatch negativity (MMN) at bilateral occipito-temporal regions in time windows 170–360 ms when no attention was paid. The MMN elicited by the fearful faces started as early as about 70–120 ms after the stimulus onset. In summary, these previous studies have found that emotional facial stimuli, especially threatening stimuli, could capture automatic attention marked by either the increased amplitude of the ERP P1, P2 and visual MMN components or the increased neural activity of the amygdala observed in fMRI scans and that this processing demonstrated a bottom-up process.

Another source of the perception of emotional faces is the top-down control of the parietal and frontal cortex (for review, see [21]). fMRI and ERP studies have also shown that frontal cortex regions modulate the sensory cortex for the processing of unattended emotional stimulus. Emotional stimuli including negative emotional facial stimuli are not processed under unattended conditions and emotional stimuli are modulated by active attention and this processing reflects a top-down process. Top-down control from the amygdala might occur when sufficient attention resources were available for fearful faces in attended trials but not in unattended trials [22]. Therefore, threatening stimuli could not capture automatic attention and the processing of facial expression appeared to be under top-down control. Two ERP studies found that a greater frontal positivity of ERP activity was generated about 100 ms after stimulus onset in response to fearful faces when the faces were attended. However, when the faces were located outside the attention focus, this emotional expression effect was completely eliminated [15], [23]. In short, the results of these studies imply that negative emotional stimuli could not be automatically processed in the unattended condition and that emotional stimuli were modulated by active attention.

How can these conflicting results be explained? According to Pessoa et al. [22], participants’ failure to modulate attention processing of emotional stimuli in the unattended condition in some studies (e.g. [22], [23], [24]) was the result of a competing task fully absorbing subjects’ attentional resources. Williams et al. [25] proposed that focusing on houses rather than on faces in Vuilleumier and colleagues’ study [15] was relatively easy and might have left residual attentional capacity for face processing in the unattended condition. Thus these explanations for the conflicting results in previous studies are based on the absence or existence of residual attentional resources in participants under the unattended condition. However, this explanation raises a practical question: how can one determine that all attentional resources have been consumed without leaving any residual attentional resources to process the emotional stimuli in the unattended condition?

To date, no appropriate method has been developed to accurately measure the distribution of attentional resources to emotional stimuli in the unattended condition. The typical research paradigm of previous studies requires participants to undergo two conditions: an attended condition in which participants are required to pay attention to the emotional stimuli directly and an unattended condition in which participants are required to pay attention to non-emotional stimuli with emotional stimuli superimposed over them. The effects of emotional stimuli on attentional processes between attended and unattended conditions is compared and indexed by behavioral measures such as reaction time and accuracy or neural activity increase of the amygdala in fMRI scans (e.g., [15]) and amplitude of the early ERP components of P1 and P2 waves (e.g., [24]). Because the level of attentional demand differed under attended and unattended conditions and emotional stimuli varied in previous studies (e.g., [22]), this kind of comparison likely produced the conflicting results.

In order to accurately measure the levels of residual attention resources that are distributed across emotional stimuli in the unattended conditions, we reasoned that the processing of these emotional stimuli in different unattended conditions should be directly compared. Therefore, the present study was designed to have all subjects perform two types of attention tasks while the same emotional facial stimuli were superimposed over the non-emotional stimuli in the unattended condition. According to Lavie’s perceptual load theory [26], which defines perceptual load as the attention resources required for attended tasks, the magnitude of attention modulation upon unattended emotional stimuli is dependent on the demands of attended tasks. This theory further states that fewer residual attentional resources would be distributed to distracters (emotional stimuli) if attention is entirely consumed by performing a highly demanding task. Conversely, on a low demand task, residual attentional resources could be available to be distributed among distracters (emotional stimuli). Based on Lavie’s perceptual load theory, our first prediction is that more attention resources will be available for the emotional stimuli under the unattended condition when the perceptual load of the attention task is low. Secondly, we predict that the interfering effect of facial expressions on the current task will decrease or disappear entirely when the perceptual load of the attention task is increased and demands full attention.

ERP recordings were found to have a better temporal response than fMRI scans in exploring the time course of attention modulation for emotional stimuli under unattended conditions [27]. Several recent studies have shown that ERP recordings reveal the time course of emotional processing effectively. The early ERP components that are involved with attention were elicited by emotional stimuli in unattended conditions [24], [27]. These findings suggest that emotional facial expressions can rapidly trigger cortical circuits that are responsive in the detection of emotionally significant events. Because the processing of emotional stimuli, especially negative emotional stimuli, generally occurs within a very short time period, and because the attention level changes quickly with the time course of emotional stimuli perception after stimuli onset, we reasoned that ERP recording would be the most appropriate method to temporally measure transient brain activity in the process of automatic attention.

The purpose of the present study was to investigate the effects of exposure to emotional face pictures on attention while performing Chinese word judgment tasks. Two experiments were conducted. In Experiment 1, subjects performed low (judging the structure of Chinese characters) and high (judging the tone) cognitive load tasks while exposed to unattended pictures of fearful, neutral, or happy faces. Their reaction time and accuracy of performance on word structure judgment were measured. We hypothesized that the reaction time for the word structure judgment would be significantly faster than for performing word tone judgment and the accuracy for performing word structure judgment would be significantly higher than for performing word tone judgment. Since more residual attention resources would be available to automatically process emotional facial stimuli while performing word structure judgment tasks than while performing word tone judgment tasks under the unattended condition, we further hypothesized that the condition of exposure to fearful faces would have a significantly longer reaction time and lower accuracy than for the conditions of exposure to neutral and happy faces while performing the word structure judgment tasks. We expected that no significant differences in reaction time and accuracy would be found among fearful, happy, and neutral faces while performing the word tone judgment tasks. In Experiment 2, subjects performed the same word judgment tasks as in Experiment 1 and their brain event-related potentials (ERPs) were measured for a period of 800 ms after the onset of the task stimulus. Similarly, we hypothesized that the amplitude of the early ERP component of the P2 wave elicited by unattended fearful faces over frontal-central-parietal recording sites would be significantly larger than that elicited by unattended neutral or happy faces while performing word structure judgment tasks and that no significant differences in P2 amplitudes would be found among fearful, happy, and neutral faces while performing word tone judgment tasks.

## Experiment 1

### Methods

#### Participants

Thirty-five healthy undergraduates and graduates (11 males and 24 females) were recruited from Beijing Normal University. Their ages ranged from 17 to 26 years old with a mean of 20.5±2.13. All subjects were right-handed as assessed with Chapman and Chapman’s scale [28]. Participants had no history of psychiatric or neurophysiological diseases and had normal or corrected vision. Each subject provided informed written consent before the experiment. The experimental procedures were approved by the Institutional Review Board of the State Key Laboratory of Cognitive Neurosciences and Learning. Twenty Yuan (RMB) was paid to each subject for participating in the experiment.

### Materials

#### Chinese words

42 high-frequency Chinese words were used in the experiment. The characters were selected from “Modern Chinese Frequency Dictionary” [29]. Each word was composed of two structural parts. Half of these Chinese words were composed with left-right structure (e.g. 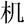), and the other half was up-down structure (e.g. 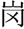). The Chinese pronunciation system is composed of four tones. There is a significant contrast in pronunciation between tone two and tone four. Tone two is a volume rising tone and tone four is a volume falling tone. Half of the Chinese words were tone two in the Chinese pronunciation tones (e.g. 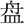), and the other half was tone four (e.g. 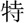). The participants viewed the same 42 words while performing word structure judgment or tone judgment. The words were presented on a monitor screen with one word for each trial. Participants were seated approximately 70 cm from the screen (no chinrest was used) so that the visual angle of the word was 2.5°.

#### Human faces

Twenty-one gray pictures of human faces were used. The faces were obtained from Ekman’s series [30] and depicted 7 individuals’ facial expressions. Each individual displayed fearful, neutral, and happy facial expressions. Four identities were female (labeled as C, MF, SW, and M), and three were male (labeled as JJ, JB, and EM). The visual angle of the face was 3.3°, face and word separated by 0.3°. Two identical faces were presented to the left and right sides of each Chinese word on every trial. The equipment used in the study was a Founder PC and a 17-inch monitor display.

### Experimental Design

A 2 (tasks: word structure vs. word tone) ×3 (emotional faces: fear, neutral, and happy) within-subjects design was adopted. Each of the six emotional conditions consisted of 42 trials. Subjects all had eight practice trials to familiarize the word structure and tone judgment tasks.

#### Tasks

Subjects were asked to perform Chinese word structure and tone judgment tasks that were irrelevant to the perception of facial expression. The faces were presented in the unattended condition while performing word structure and tone judgment tasks. For the word structure judgment task, subjects were instructed to assess whether each word had a left-right structure or up-down structure by pressing ‘z’ or ‘/’ on the keyboard as quickly as possible. For the word tone task, subjects were asked to make their response about whether each word was tone two or tone four in the Chinese pronunciation tone system by pressing the corresponding buttons on the keyboard as quickly as possible. These buttons were also counterbalanced on the keyboard. The presentation order of the two tasks was counterbalanced between subjects.

### Procedures

The experimental procedures were programmed with E-Prime 1.1 software (Psychology Software Tools Inc: www.pstnet.com/eprime). Participants were tested individually by sitting on a chair at a distance of 80 cm away from the computer screen in a well-lit room. All stimuli were presented visually on a white against black background at the center of the screen. A fixation mark was first presented in the center of the screen for 500 msec. Then a Chinese word was displayed in the center of the screen for 100 msec with two identical human faces shown on each side of the Chinese word. The subject was asked to pay attention to the Chinese word and ignore the human faces. The presentation of fearful, neutral, or happy faces with the Chinese word was randomly distributed. After that, there was a 1900 ms blank time for the subject to judge the word structure or word tone. The subject was asked to make a response by pressing the corresponding buttons as described earlier, and the interval between two trials ranged from 250 to 850 msec. The subject’s reaction time and correct judgment rate were measured electronically.

## Results

A total of 35 subjects’ experimental data were collected and analyzed. Table 1 presents the means and standard deviations of the reaction time to complete the word structure and word tone judgment tasks when fearful, neutral and happy faces were unattended respectively. As can be seen in Table 1, subjects had a much shorter reaction time while performing word structure judgment tasks than while performing word tone judgment tasks.

**Table 1:** Means and standard deviations of the reaction time (ms) for the word structure (WS) and word tone judgment tasks (WT) when fearful, neutral and happy faces were unattended respectively.

RT	Fearful(Mean & SD)	Neutral(Mean & SD)	Happy(Mean & SD)
Task of WS	637.93(14.2)	622.74(13.57)	633.12(13.87)
Task of WT	792.86(19.14)	795.34(20.09)	792.11(19.32)

A 2 (tasks: word structure vs. word tone) ×3 (emotional faces: fear, neutral, and happy faces) within subjects repeated measures analysis of variance (ANOVA) on reaction time was performed. The results showed that the main effect of type of task was significant (F_(1, 34)_ = 135.223, P<0.0001). The mean reaction time for word structure judgment was significantly faster than that for word tone judgment, indicating that more time was needed to perform word tone judgment tasks. No significant main effects for emotional faces (F_(1, 34)_ = 2.178, P = 0.126, ε = 0.915) were found. There was a significant interaction effect between tasks and emotional faces (F_(2, 68)_ = 3.961, P = 0.031, ε = 0.836). Further analysis showed that there were significant differences in the reaction time among the conditions of exposure to fearful, neutral, and happy faces while performing the word structure judgment tasks (F = 5.24, P = 0.008). The paired-comparisons showed that the mean reaction time for the condition of exposure to fearful faces was significantly longer than that for the condition of exposure to neutral faces (t = 2.599, P = 0.014, LSD = 15.18 ms) and that the mean reaction time for the condition of exposure to happy faces was significantly longer than that for the condition of exposure to neutral faces (t = 2.569, P = 0.015, LSD = 10.38 ms). However, there were no significant differences in mean reaction time between exposure to fearful and happy faces. Further analysis showed that there were no significant differences in the mean reaction time among the conditions of exposure to fearful, neutral, and happy faces while performing the word tone judgment tasks.

Table 2 presents the means and standard deviations for accuracy in performing the word structure and word tone judgment tasks when fearful, neutral and happy faces were superimposed respectively. A 2 (tasks: word structure vs. word tone judgment task) × 3 (emotional faces: fearful, neutral, and happy faces) within subjects repeated measures ANOVA on correct percentage was performed. The results showed a significant main effect of type of task (F_(1, 34)_ = 38.796, P<0.0001). The mean correct percentage of the word tone judgment task was significantly lower than that of the word structure judgment task, indicating that it was more difficult to complete the word tone judgment task than the word structure judgment task. No significant main effects for emotional faces were found (F_(2, 68)_ = 0.657, P = 0.517, ε = 0.966). There was a significant interaction of correct percentage between tasks and emotional faces (F_(2, 68)_ = 3.934, P = 0.028, ε = 0.908). Further analysis showed that there were significant differences in the correct percentage rate among the conditions of exposure to fearful, neutral, and happy faces while performing the word structure judgment tasks (F = 3.43, P = 0.038). The paired-comparisons showed that the mean correct percentage for the condition of exposure to fearful faces was significantly lower than that for the condition of exposure to happy faces (t = 2.662, P = 0.012, LSD = 0.0177) and that the mean correct percentage for the condition of exposure to happy faces was marginally significantly higher than that for the condition of exposure to neutral faces (t = 2.026, P = 0.051, LSD = 0.014). No significant difference was found in the mean correct percentage between neutral and fearful faces. There were no significant differences in the mean correct percentage among the conditions of exposure to fearful, neutral, and happy faces while performing the word tone judgment tasks.

**Table 2:** Means and standard deviations of the correct percentage (1 = 100%) for the word structure (WS) and word tone judgment tasks (WT) when fearful, neutral and happy faces were unattended respectively.

Correct	Fearful(Mean & SD)	Neutral(Mean & SD)	Happy(Mean & SD)
Task of WS	0.971(0.005)	0.967(0.008)	0.953(0.007)
Task of WT	0.883(0.014)	0.897(0.01)	0.901(0.013)

## Experiment 2

### Methods

#### Participants

Twenty-six (14 males, 12 females) healthy undergraduates and graduates were recruited from Beijing Normal University. The data from two participants was not included in the analysis because of an EMG artifact. Ages ranged from 17 to 25 years old with a mean of 20.8. None of the participants was from Experiment 1.

All subjects were right-handed as assessed with Chapman and Chapman’s scale [28]. All subjects were healthy with no past history of psychiatric or neurological diseases and had normal or corrected-to-normal vision. Each subject provided informed written consent before the experiment. The experimental procedures were approved by the Institutional Review Board of the State Key Laboratory of Cognitive Neurosciences and Learning. Fifty Yuan (RMB) was paid to each subject for participating in the experiment.

### Materials

The same materials were used as in Experiment 1.

### Apparatus

A Founder PC and a 17-inch monitor display were used. In addition, a NeuroSCAN system using a 64-channel Quick-cap with Ag-AgCl electrodes (Compumedics, Melbourne, Australia) was used.

### Experiment Design

The same experiment design as in Experiment 1 was employed except that there were 84 trials for each condition.

#### Tasks

The same tasks as in Experiment 1 were used.

### Procedures

The same procedures as in Experiment 1 were used except that ERP recordings were measured while performing word structure or word tone judgment tasks. Scalp voltages were recorded by a NeuroSCAN system and using a 64-channel Quick-cap arranged according to the international 10–10 system (Compumedics, Melbourne, Australia). Eye movements were recorded using horizontal and vertical electrooculograms (HEOG and VEOG). Sixty-two channels were recorded with a linked mastoids reference. Electrode impedance was kept below 5 kΩ. The amplifier band pass range was 0.05–100 Hz. EEG and EOG signals were sampled at 500 Hz.

Following electrode placement, the subject was individually seated in a comfortable chair in a dimly lit testing room and informed of the experimental tasks. To reduce muscle artifacts in the EEG recordings, the subject was instructed to assume a comfortable position and avoid body movement and unnecessary eye blinks. The presence of an adequate EEG signal was determined by visual inspection of the raw signal on the computer display.

### ERP Offline Analysis

ERP analyses were conducted offline. Ocular artifact processing for each participant was performed for each epoch by a correction algorithm (Semlitsch, 1986). The EEG data were divided into epochs of 900 ms, starting from 100 ms before stimulus onset and ending 800 ms after it. Trials with incorrect responses were excluded from the epochs. The pre-stimulus baseline level used was the mean activity from 100 to 0 ms before stimulus onset. After baseline correction, the EEG data were low-pass filtered below 45 Hz (24 dB/oct). Epochs exceeding the range of −75 to 75 µV were rejected as artifacts. The remaining trials were averaged for each of the tasks (word structure vs. word tone) and for each kind of facial expression (fearful, neutral, and happy) separately for each subject. The number of valid trials that were used for averaging ranged from 50 to 84 trials (mean = 76.3 trials). After the inspection of the grand average waveform and the difference between the conditions of fearful faces and neutral faces, we analyzed the frontocentral P2 component (mean amplitudes between 154–190 ms after stimulus onset).

### Behavioral Results

Two subjects’ ERP data were excluded due to significantly large recording artifacts. A total of 26 subjects’ experimental data were collected and statistically analyzed. Table 3 presents the means and standard deviations of the reaction time to complete the word structure and word tone judgment tasks when fearful, neutral and happy faces were unattended respectively.

**Table 3:** Means and standard deviations of the reaction time (ms) in Experiment 2 for the word structure and word tone judgment tasks when fearful, neutral and happy faces were unattended respectively.

RT	Fearful(Mean & SD)	Neutral(Mean & SD)	Happy(Mean & SD)
Task of WS	638.869(18.823)	630.53(17.59)	638 (18.679)
Task of WT	749.002(27.966)	755.927(27.43)	751.832(27.01)

Note: WS refers to word structure judgment task and WT refers to word tone judgment task.

A 2 (tasks: word structure vs. word tone) ×3 (emotional faces: fear, neutral, and happy faces) within subjects repeated measures analysis of variance (ANOVA) on reaction time was performed. The results showed that the main effect of type of task was significant (F_(1, 25)_ = 19.397, P<0.001). The mean reaction time for word structure judgment was significantly faster than that for word tone judgment, indicating that more time was needed to perform word tone judgment tasks. No significant main effects for emotional faces (F_(2, 50)_ = 0.227, P = 0.762, ε = 0.848) were found. The interaction of task and emotion was significant (F_(2, 50)_ = 6.409, P = 0.004, ε = 0.942). Simple effect analysis showed that there were significant differences in the reaction time among the three types of emotional faces while performing the word structure judgment tasks (F = 3.29, P = 0.045). The paired-comparisons showed that the mean reaction time for the condition of exposure to fearful faces (t = 2.575, P = 0.016, LSD = 8.339 ms) or happy faces (t = 2.190, P = 0.038, LSD = 7.469 ms) were significantly longer than that for the condition of exposure to neutral faces.

Table 4 presents the means and standard deviations of correct percentage while performing the word structure and word tone judgment tasks when fearful, neutral and happy faces were superimposed respectively. A 2 (tasks: word structure vs. word tone judgment task) × 3 (emotional faces: fearful, neutral, and happy faces) within subjects repeated measures ANOVA on correct percentage was performed. The results showed that the main effect of type of tasks was not significant (F_(1, 25)_ = 2.177, P = 0.153). No significant main effects for emotional faces were found (F_(2, 50)_ = 0.684, P = 0.483, ε = 0.822). There was also no significant interaction of correct percentage between tasks and emotional faces (F_(2, 50)_ = 0.323, P = 0.724, ε = 0.993).

**Table 4:** Means and standard deviations of the correct percentage (1 = 100%) in Experiment 2 for the word structure and word tone judgment tasks when fearful, neutral and happy faces were unattended respectively.

Correct percent	Fearful(Mean & SD)	Neutral(Mean & SD)	Happy(Mean & SD)
Task of WS	0.965(0.008)	0.97(0.007)	0.963(0.009)
Task of WT	0.938(0.21)	0.937(0.20)	0.934(0.21)

Note: WS refers to word structure judgment task and WT refers to word tone judgment task.

### ERP Results

**Figure 1:**
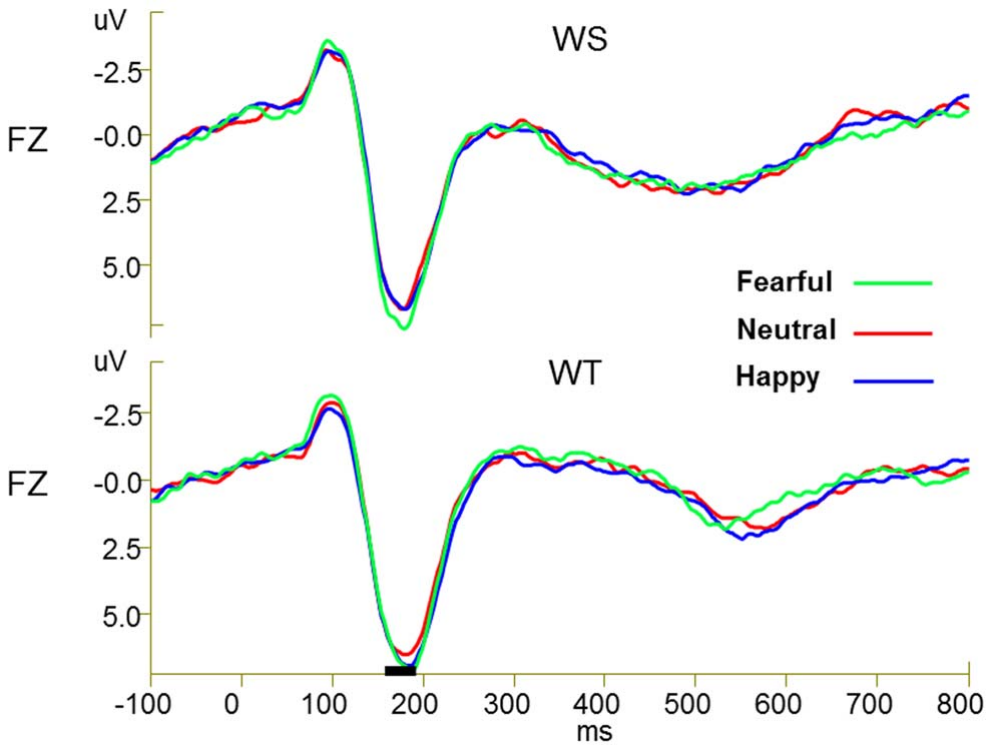
Grand-average ERPs by six conditions at FZ. Grand-averaged event-related potential waveforms elicited by fearful, neutral, and happy faces respectively during performance of word structure tasks (WS) and word tone tasks (WT) at FZ electrode.

**Figure 2:**
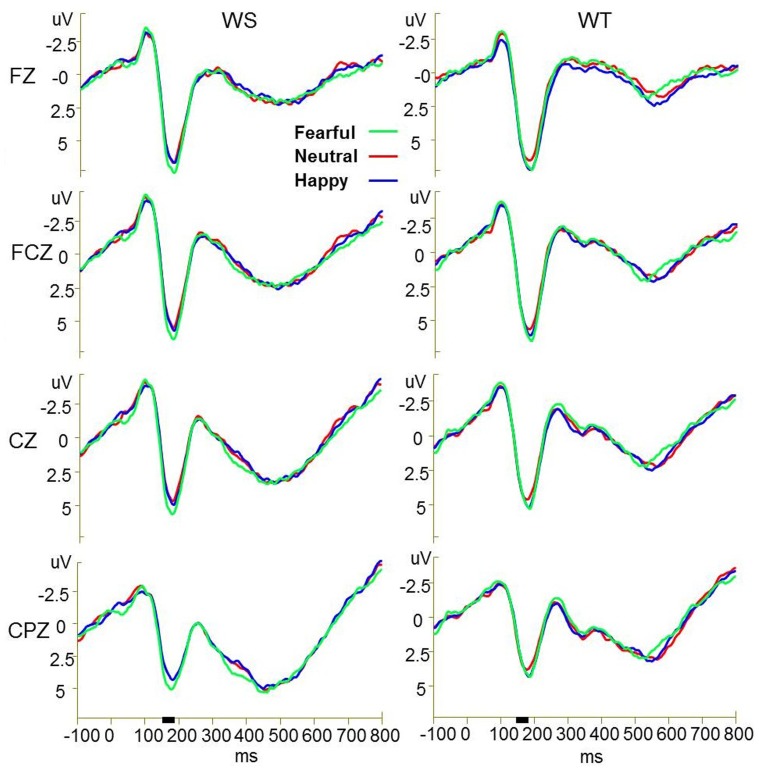
Grand-average ERPs by six conditions at FZ, FCZ, CZ, CPZ electrodes. Grand-averaged event-related potential waveforms elicited by fearful, neutral, and happy faces respectively during performance of word structure tasks (WS) and word tone tasks (WT) at FZ, FCZ, CZ, CPZ electrode.

**Figure 3:**
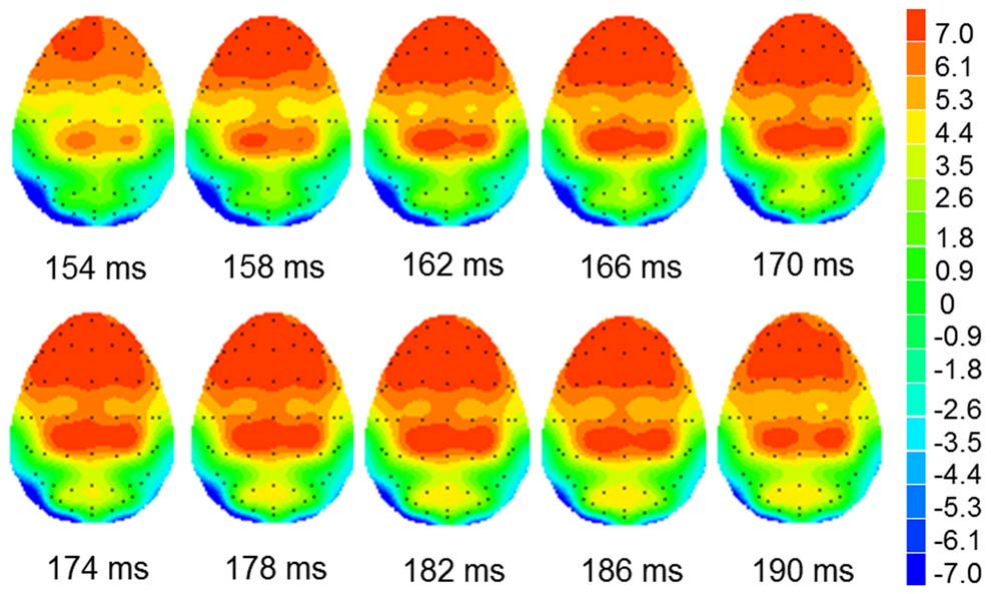
Topography of fearful faces at time window of 154–190 ms. Scalp potential maps reveal the topography of the interference effects of fearful faces on the Chinese word judgment tasks in the time window of 154–190 ms.

Two subjects’ ERP data were excluded due to significantly large recording artifacts. Therefore, a total of 26 subjects’ ERP data were statistically analyzed. Figure 1 and Figure 2 presents the grand-averaged event-related potential waveforms elicited by fearful, neutral, and happy faces respectively during performance of word structure judgment and word tone judgment tasks. The augmented P2 amplitudes over frontal-central-parietal electrodes in response to fearful faces compared with happy and neutral faces developed around 154 ms after stimulus onset and were most pronounced around 176 ms post-stimulus while performing the word structure judgment task. However, the augmentation in the P2 amplitude in response to fearful faces was not observed while performing the word tone judgment task. Figure 3 presents scalp potential maps which reveal the topography of the interference effects of fearful faces on the Chinese word judgment tasks in the time window of 154–190 ms. As can be seen in Figure 3, the increase in scalp potentials over frontal-central-parietal electrodes elicited by the exposure to fearful faces while performing Chinese word judgment tasks is most significant in the time window of 154 to 190 ms.

Table 5 describes the averaged P2 amplitudes at electrode FZ elicited by three categories of facial expressions in the word structure judgment and word tone judgment tasks respectively. Statistical analysis was conducted within factors on post-stimulus time intervals based on the grand-averaged event-related potential P2 waveforms (154–190 ms). A 2 (tasks: word structure vs. word tone) ×3 (emotional faces: fearful, neutral and happy) ×20 (electrodes: FZ, F1, F2, F3, F4, FCZ, FC1, FC2, FC3, FC4, CZ, C1, C2, C3, C4, CPZ, CP1, CP2, CP3, CP4) within-subjects repeated measures ANOVA was performed on the ERP components of P2. The results showed that the main effect of emotional faces on P2 amplitude was significant (F_(2, 50)_ = 4.026, p = 0.028, ε = 0.915) and the main effect of electrode site on P2 amplitude was also significant (F_(19, 475)_ = 17.869, P<0.001, ε = 0.103). The largest amplitude of P2 was generated at Fz electrodes (M = 6.713 µv) with the exposure to fearful faces in the word structure judgment task. However, the main effect of task on P2 amplitude was not significant (F_(1, 25)_ = 0.0002, p = 0.988, ε = 0.915). No significant differences were found in P2 amplitudes between fearful and happy faces and between happy and neutral faces. In addition, an interaction effect was found between word tasks and emotional face stimuli (F_(2, 50)_ = 3.367, p = 0.048, ε = 0.907). Further comparisons indicated that there were significant differences in P2 amplitudes among the conditions of exposure to fearful, neutral, and happy faces while performing the word structure judgment task (F_(2, 50)_ = 7.397, P = 0.002, ε = 0.949). Paired comparisons indicated that the P2 amplitude elicited by fearful faces was significantly larger than that by neutral faces (F = 12.677, p = 0.002, LSD = 0.746) or happy faces (F = 12.427, p = 0.002, LSD = 0.74) respectively. There was no significant difference between the P2 amplitudes elicited by neutral or happy faces. However, further comparisons indicated that there were no significant differences in P2 amplitudes among the conditions of exposure to fearful, neutral, and happy faces in the word tone judgment task. It was interesting to note that the exposure to fearful faces elicited the highest P2 amplitude while performing the word structure judgment task (see Table 5).

**Table 5:** Means and standard deviations of P2 mean amplitudes (µV) in the time window of 154–190 ms at electrode FZ in Experiment 2 for the word structure and word tone judgment tasks when fearful, neutral and happy faces were unattended respectively.

P2 amplitudes	Fearful(Mean & SD)	Neutral(Mean & SD)	Happy(Mean & SD)
Task of WS	6.713(0.772)	6.001(0.738)	5.995(0.764)
Task of WT	6.394(0.861)	6.199(0.821)	4.427(0.742)

Note: WS refers to word structure judgment task and WT refers to word tone judgment task.

## Discussion

The aim of the present two experimental studies was to investigate the automatic processing of emotional facial expressions while performing the low (word structure judgment tasks) or high (word tone judgment tasks) demand cognitive tasks under unattended conditions. In Experiment 1, we investigated the effects of exposure to unattended fearful, neutral or happy faces on behavioral measurements such as reaction time and accuracy while performing word structure and tone judgment tasks. The results indicate that the automatic processing was indexed by the interfering effects of exposure to emotional faces and that the automatic processing of emotional faces was triggered while performing the word structure judgment tasks, but not while performing the word tone judgment tasks under the unattended conditions. The experimental results support our hypothesis that reaction time for word structure judgments would be significantly faster than for word tone judgments and accuracy would be significantly higher. These findings indicate that the word structure judgment task was easier than the word tone judgment task.

Chinese characters have independent graphic and phonology information. Chen et al. [31], [32] found that for high frequency Chinese characters, the graphic component was first activated and then the semantics and that the phonology needs the longest time to process. However, for low frequency Chinese characters, the first activation still was graphic; then semantics and phonology are processed simultaneously. Our experimental findings are consistent with Chen et al’s studies. The reaction time was shorter for performing the word structure judgment task (low cognitive load) than for performing the word tone judgment task (high cognitive load). The hypothesis that the condition of exposure to fearful faces would have a significantly longer reaction time and lower correct percentage of performance than for the conditions of exposure to neutral and happy faces while performing the word structure judgment tasks was partially supported. We found that subjects in the condition of exposure to fearful faces had a significantly slower reaction time than in the condition of exposure to neutral faces and subjects in the condition of exposure to fearful faces had a significantly lower correct percentage than in the condition of exposure to happy faces. These results demonstrate that exposure to fearful faces interfered with the performance of the low demand cognitive tasks but not the high demand cognitive tasks under unattended conditions. The finding that negative emotional faces were more likely to interfere with the performance of tasks is consistent with several previous studies (e.g., [4], [10], [11], [13]).

The results of Experiment 1 reveal that the priority of processing the unattended fearful faces depends on the level of demanded perceptual load by the target task. We believe that attention bias occurred for the fearful faces because they attracted more attention resources than neutral faces while performing the low-perceptual load task (word structure judgment). However, this phenomenon was not observed while performing the high-perceptual load task (word tone judgment). These results verified that perceptual load influenced the priority of processing for unattended emotional stimuli. Also, our experimental results support Harris & Pashler’s [33] theoretical speculation that perceptual analysis of high-priority stimuli is subject to the capacity limitations for other stimuli: when enough capacity is available for a high-priority stimulus to be perceived, a transient surprise reaction may interrupt ongoing processing.

In Experiment 2, we tested if ERP recordings would display a characteristic pattern of the automatic processing of unattended emotional faces while performing word structure and tone judgment tasks. The ERP results support our hypothesis that the amplitude of the early ERP component of the P2 wave elicited by unattended fearful faces would be significantly larger than that elicited by unattended neutral or happy faces while performing word structure judgment tasks. We found that the enhanced P2 amplitude was elicited by fearful faces in the time window of 154 ms to 190 ms after stimuli onset while performing the low-perceptual load task but not while performing the high-perceptual load task. Previous studies found that component of ERP P2 marks the neurophysiological process of automatic attention and that P2 peaking approximately at 150 ms was associated with attention orientation [34], [35], [36], [37]. Recent research further indicates that the P2 component displayed the highest amplitude when deviant stimulus was presented. Carretié [27] found that the P2 component showed greater amplitudes in response to emotionally deviant stimuli (negative and positive) than in response to neutral deviant stimuli. Huang and Luo [38] also found that the amplitude of the P2 component evoked by negative pictures was significantly larger than by neutral pictures. Thomas, Johnstone and Gonsalvez [39] reported that compared to neutral words, words with threatening content were associated with larger P2 amplitude in the right hemisphere than in the left hemisphere.

In the present study, we observed that fearful faces enhanced P2 amplitude that peaked at 160 ms and was distributed over frontal-central electrodes. This finding is consistent with those reported in previous studies. Eimer, Holmes and McGlone [40] found that emotional faces elicited a broadly distributed positive peak as compared with neutral faces and that the positive peak appeared from 180 ms to 220 ms after stimulus onset in the frontal and central sites. Similar findings were replicated by Holmes, Kiss and Eimer [41] with P2 enhanced at 220 ms after the stimulus onset. Ashley, Vuilleumier, and Swick [42] provided further evidence that upright fearful expressions enhanced the frontocentral P200 component. Those findings indicated that the enhanced frontocentral P2 wave might be a neurological index of the automatic detection of emotions and that the early stage of fearful expression effect reflects the pre-attentive automatic assessment of emotional faces [43].

The characteristic ERP patterns with exposure to fearful faces in Experiment 2 were very well matched with the finding from the behavioral measurements in Experiment 1. Our postulation is that when residual attention resources were available, such as while performing a low cognitive demand task, exposure to unattended fearful faces could automatically capture attention resources as demonstrated by a longer reaction time and lower correct percentage of performance. This ERP pattern was not observed with exposure to happy faces in unattended conditions while performing both the low or high demand cognitive tasks. These findings demonstrated that negative emotions such as fearful faces affect automatic attention processing more significantly than do positive or neutral facial expressions and again provides biological evidence for the previously proposed evolutionary explanation that the ability to perceive threatening stimuli automatically provides an evolutionary advantage in warning creatures of potential danger and therefore plays an important survival role [1]. Furthermore, it shows that this kind of orienting response is realized through the mechanism of automatic attention, which is an unconscious stimulus-driven bottom-up process [4].

However, our findings of automatic processing in response to exposure to unattended fearful faces while performing the low-perceptual load task (word-structure judgment) are not consistent with a previous study’s report [23] in which researchers did not find the automatic processing effect of negative facial expressions in the unattended condition. One possible explanation for this inconsistency between the two studies could be that the task that was used in their study was more difficult than the task of word-structure judgment and was similar to the task of word-tone judgment in our study. Therefore, no residual attention resources were available in their study to process unattended fearful stimuli automatically.

The behavioral measurement in the present study also revealed that compared to exposure to neutral faces, exposure to happy faces elicited a longer reaction time when subjects were performing the word structure judgment task. This attention bias disappeared when performing the word tone judgment task. Our finding is consistent with a recent study in which researchers found that, as a distractor, a happy face slowed down the target detection process [44]. Another recent study reported that happy faces had the advantage in capturing attention, even outside of overt visual attention [45]. Although happy faces mirrored fearful faces in behavioral measurement in the present study, they might trigger a mechanism that was different from fearful faces in the process of eliciting behavioral effects on attentional performance. Exposure to happy faces might enhance positive mood, which in turn induces positive attention bias. A recent study, using the flanker task, found that the positive attention bias impaired the inhibition of flanker distraction, through increasing the scope of spatial attention [46]. The positive effect might enhance attention to the flanker by enlarging the breadth of attention allocation and then absorbing more unattended information [47].

However, ERP P2 changes in the present study were not associated with the behavioral pattern of attention bias from exposure to happy faces. It has been found that fearful faces or threatening pictures always activated the amygdala and that happy faces did not (1, 16, 21). Kesler-West et al [48] reported that angry or happy faces activated the anterior cingulate, the orbitofrontal, and inferior prefrontal cortices. The ERP P2 did not show the difference between exposure to happy faces and exposure to neutral faces under low or high-perceptual load tasks in the present study. It might be that happy faces captured attention resources in difference ways with different neuronal networks and needed different attention resources.

In conclusion, the present study found that exposure to fearful faces while performing low demand cognitive tasks under unattended conditions interfered with the performance of the tasks as demonstrated by a longer reaction time and lower performance accuracy. This phenomenon was verified by the characteristic increase of P2 amplitude in ERP recordings in response to exposure to unattended fearful faces while performing low demand cognitive tasks. Our findings provide empirical and biological evidence to support the theoretical speculation that unattended threatening stimuli are automatically processed while performing low (word structure judgment tasks) but not while performing high (word tone judgment tasks) perceptual load tasks.
